# Efficacy differences and predictors of personalized mixed acoustic therapy in chronic tinnitus patients with and without hearing loss

**DOI:** 10.3389/fneur.2026.1889942

**Published:** 2026-07-01

**Authors:** Yuan Chen, Yue Ba, Meiling Sun, Jun Hu, Qin Jiang, Bin Liu

**Affiliations:** 1 Department of Geriatrics, The Affiliated Brain Hospital of Nanjing Medical University, Nanjing Brain Hospital, Nanjing, Jiangsu Province, China; 2 School of Basic Medical Sciences, Nanjing Medical University, Nanjing, Jiangsu Province, China; 3 Department of Radiology, The Affiliated Brain Hospital of Nanjing Medical University, Nanjing Brain Hospital, Nanjing, Jiangsu Province, China

**Keywords:** chronic tinnitus, hearing loss, mixed acoustic therapy, personalized medicine, tinnitus handicap inventory

## Abstract

**Objective:**

This study aimed to evaluate the efficacy of a personalized mixed acoustic therapy (narrow-band noise combined with music) in patients with chronic tinnitus, comparing those with and without hearing loss (HL vs. NHL), and to identify baseline predictors of treatment response.

**Methods:**

A total of 111 patients with chronic tinnitus were enrolled (HL group, *n* = 51; NHL group, *n* = 60). At baseline, all participants underwent bilateral subjective tinnitus acoustic assessment and completed the HAMA, HAMD, PSQI, and THI scales. The same assessments were repeated after a 3-month mixed acoustic intervention.

**Results:**

At baseline, the NHL group was younger and had a higher prevalence of psychiatric history and insomnia than the HL group. After 3 months, both groups showed comparable improvements in tinnitus frequency, loudness, and PSQI scores (significant main effect of time, no group × time interaction). However, the NHL group showed significantly greater reductions in HAMA and THI scores (significant group × time interaction; both *p* < 0.05). Binary logistic regression identified NHL status, female sex, and lower baseline PSQI and HAMA scores as independent predictors of a favorable outcome (THI reduction ≥7).

**Conclusion:**

Personalized mixed acoustic therapy improves tinnitus perception and sleep quality in both HL and NHL patients, but provides significantly greater psychological benefit in patients without hearing loss. The bilateral assessment protocol also accommodated patients with central “head noise” who could not lateralize their tinnitus. These findings support a stratified approach to tinnitus management based on audiometric and psychometric phenotypes.

## Introduction

Chronic tinnitus, the persistent perception of sound in the absence of an external stimulus, is a substantial clinical burden that impairs quality of life, emotional well-being, and sleep across a large segment of the adult population, particularly older adults. Although tinnitus is frequently associated with hearing impairment, the relationship between the two is complex and heterogeneous: severe hearing loss does not always lead to tinnitus, and disabling tinnitus often occurs in individuals with normal audiometric thresholds (1, 2). This variability suggests that distinct pathophysiological mechanisms operate across different patient subgroups. A further clinical challenge is that many patients cannot distinguish between peripheral “tinnitus” (localized to one or both ears) and central “head noise” (perceived diffusely within the head). Despite the high prevalence of this presentation, patients who cannot lateralize their tinnitus to a specific ear remain understudied, representing an important gap in our understanding and treatment of this common condition.

Current management of chronic tinnitus includes sound therapy, cognitive behavioral therapy (CBT), pharmacological treatment, and neuromodulation (3). Among these, sound therapy is a foundational option because it is non-invasive and has a favorable safety profile (4). Conventional techniques such as masking and notched music therapy aim to normalize maladaptive neural firing along the auditory pathway (5). However, their efficacy—particularly for tinnitus-related anxiety, depression, and functional disability—remains inconsistent and is often inadequate, especially in patients with coexisting hearing impairment. Recent prospective work has further shown that personalized tinnitus masking guided by pure-tone audiometry can yield measurable clinical benefit, supporting audiometry-driven customization of acoustic therapy (6). This gap highlights the need for more precise acoustic strategies that address both the perceptual and emotional aspects of tinnitus, tailored to individual patient characteristics—particularly the audiometric profile (7, 8).

Novel mixed acoustic protocols that combine narrow-band noise with music offer a dual-action mechanism: the noise component may suppress aberrant neural synchrony in the auditory cortex, while the musical component engages limbic and reward circuits to modulate emotional responses (5, 9). Despite this promise, direct comparisons of such mixed therapies in patients with versus without hearing loss are scarce. In particular, it remains unclear how hearing status influences treatment outcomes, both in psychoacoustic parameters and in psychological well-being (10, 11). Identifying baseline predictors of treatment response is also important for advancing personalized care, but this question remains under-investigated.

To address these knowledge gaps, we conducted a prospective study of personalized mixed acoustic therapy in middle-aged and older adults with chronic tinnitus. This study makes three specific contributions to the field. *First*, we directly compare the psychological efficacy of mixed acoustic therapy in patients with versus without hearing loss within a single prospective cohort—a head-to-head comparison that, to our knowledge, remains under-investigated. *Second*, we apply a bilateral subjective acoustic assessment protocol that quantifies tinnitus frequency and loudness in each ear independently, thereby enabling the inclusion of patients who cannot lateralize their tinnitus perception (“head noise”), a clinically prevalent but understudied subgroup. *Third*, we identify baseline predictors of favorable response using binary logistic regression, providing an empirical basis for pre-treatment patient selection in routine practice.

## Methods

### Study design and ethical considerations

This prospective observational cohort study was conducted at the Affiliated Brain Hospital of Nanjing Medical University. The primary aim was to evaluate a personalized mixed acoustic therapy (narrow-band noise combined with music) in patients with chronic tinnitus, stratified by hearing loss status. The study was approved by the institutional Ethics Committee (approval no. 2025-KY006-1), and written informed consent was obtained from all participants before any study procedures. Patients were enrolled between January and December 2025.

### Participants

A total of 111 patients with chronic tinnitus were enrolled. Based on pure-tone audiometry, participants were classified per the 1997 WHO classification of hearing impairment (12) into a hearing loss group (HL; *n* = 51) and a non-hearing-loss group (NHL; *n* = 60).

### Inclusion and exclusion criteria

Inclusion criteria were: (1) chronic subjective tinnitus diagnosed according to the 2014 clinical practice guideline of the American Academy of Otolaryngology–Head and Neck Surgery (13); (2) age 40–60 years; (3) ability and willingness to undergo cranial magnetic resonance imaging (MRI); (4) no current use of psychotropic medication; (5) no participation in any other interventional study within the preceding 3 months; and (6) intact consciousness with expected adherence to the full treatment and assessment schedule.

Exclusion criteria were: (1) failure to meet any inclusion criterion; (2) mean pure-tone audiometry (PTA) threshold >60 dB HL; (3) intracranial abnormalities on MRI that could account for tinnitus (cerebral infarction, encephalomalacia, internal jugular vein compression, or arteriovenous malformation); (4) neurodegenerative or neurological disease that could affect cognition or assessment validity (cognitive impairment, Parkinson’s disease, epilepsy, or multiple system atrophy); (5) acute or active disease of the external, middle, or inner ear within the preceding 3 months; or (6) chronic comorbidity including hypertension, diabetes mellitus, or coronary artery disease.

### Baseline assessments

At enrollment, each participant completed the following baseline assessments.

#### Demographics and medical history

Age, sex, and detailed medical history were recorded, with particular attention to prior psychiatric diagnoses and chronic insomnia.

#### Audiological and tinnitus psychoacoustic evaluation

All participants underwent a complete audiological workup. Pure-tone audiometry (PTA) was used to define hearing thresholds and assign group membership. Otoscopy and tympanometry were performed to exclude middle ear disorders. Tinnitus psychoacoustic properties were measured using a dedicated testing and fitting platform (Tinifit 7.0.0 M, Foshan Bozhi). For each ear, the platform identified the perceived tinnitus frequency (Hz) and the relative tinnitus loudness, defined as the difference between the hearing threshold at the tinnitus frequency and the perceived tinnitus loudness, expressed in dB sensation level (SL). The ear with the greater relative loudness was designated the “ipsilateral” side and the contralateral ear the “opposite” side. Bilateral sound feedback and residual inhibition tests were also performed on this system.

#### Neuroimaging

All participants underwent non-contrast brain magnetic resonance imaging (MRI) and magnetic resonance angiography (MRA) to screen for the structural and vascular abnormalities listed in the exclusion criteria.

#### Psychometric assessments

Trained clinicians administered the following validated instruments: the Hamilton Anxiety Rating Scale (HAMA) (14), the Hamilton Depression Rating Scale (HAMD) (15), the Tinnitus Handicap Inventory (THI) (16, 17), and the Pittsburgh Sleep Quality Index (PSQI) (18), the last reflecting sleep quality over the preceding month.

### Intervention: mixed acoustic therapy

A personalized mixed acoustic protocol was delivered using the Tinifit system. After baseline tinnitus characterization, the system’s acoustic encoding software generated an individualized “composite acoustic prescription” combining narrow-band noise centered on each patient’s tinnitus frequency with calming musical content. The musical content comprised natural ambient sounds (e.g., rainfall) and Chinese classical pieces performed on the *guzheng*, a traditional stringed instrument. The prescription algorithm took into account each patient’s hearing thresholds and tinnitus psychoacoustic parameters (frequency and loudness). The acoustic mixture was delivered through calibrated headphones. After a trial session to confirm comfort and tolerability, participants used the therapy daily for 3 h, over three consecutive months. Adherence was monitored using patient diaries and device usage logs.

### Outcome measures and follow-up

Primary outcomes were changes in tinnitus psychoacoustics (frequency and relative loudness) and psychometric scores from baseline to the 3-month follow-up. The post-intervention assessment mirrored the baseline protocol: bilateral tinnitus frequency and loudness were re-measured with the Tinifit system, and the HAMA, HAMD, PSQI, and THI were re-administered. For the predictor analysis, a “favorable outcome” was prospectively defined as a reduction of ≥7 points in the total THI score from baseline to the 3-month follow-up, in line with the established minimal clinically important difference for the THI (19); a reduction of <7 points was defined as an “unfavorable outcome” (20).

### Statistical analysis

All analyses were performed in SPSS (version 16.0; SPSS Inc., Chicago, IL, USA). Continuous variables are presented as mean ± standard deviation (SD) and categorical variables as frequency (percentage) (21). Normality was assessed with the Shapiro–Wilk test. For baseline comparisons, independent-samples *t*-tests were used for continuous variables and *χ*^2^ tests for categorical variables.

To evaluate the effect of the intervention across groups and time, a 2 (Group: HL vs. NHL) × 2 (Time: baseline vs. 3 months) repeated-measures analysis of variance (RM-ANOVA) was performed for each continuous outcome (tinnitus frequency, loudness, HAMA, HAMD, PSQI, and THI). A significant main effect of time would indicate an overall pre-to-post change; a significant Group × Time interaction would indicate that the change over time differed between the HL and NHL groups. Significant interactions were further decomposed using simple-effect analyses.

To identify baseline predictors of a favorable outcome (THI reduction ≥7 points), we performed binary logistic regression. Candidate predictors were group (HL/NHL), age, sex, history of psychiatric disorder, history of insomnia, and baseline tinnitus frequency, loudness, HAMA, HAMD, and PSQI scores. Results are reported as odds ratios (OR) with 95% confidence intervals (CI). All tests were two-sided, with *p* < 0.05 considered statistically significant.

## Results

### Baseline clinical characteristics

The cohort comprised 111 patients with chronic tinnitus, classified into an HL group (*n* = 51) and an NHL group (*n* = 60) as described above (Table 1). At baseline, NHL patients were significantly younger than HL patients (*t* = 4.88, *p* < 0.05), had a higher prevalence of prior psychiatric disorders (*χ*^2^ = 26.60, *p* < 0.05), and reported more frequent histories of insomnia (*χ*^2^ = 49.20, *p* < 0.05). Perceived tinnitus frequency on the ipsilateral side was significantly lower in the NHL group than in the HL group (*t* = 2.904, *p* = 0.004). No significant between-group differences were observed for sex, relative tinnitus loudness, or baseline HAMA, HAMD, PSQI, or THI scores.

**Table 1:** Demographic and clinical characteristics of the two groups before intervention.

Variable	Group 1 (NHL, *n* = 60)	Group 2 (HL, *n* = 51)	t/*χ*^2^	*p*
Age (years)	49.00 ± 5.97	54.84 ± 6.65	*t* = 4.88	<0.05**
Sex (male, *n* %)	23 (38.33)	25 (49.02)	*χ*^2^ = 0.88	0.347
History of psychiatric disorder (*n* %)	41 (68.33)	9 (17.65)	*χ*^2^ = 26.60	<0.05**
History of insomnia (*n* %)	57 (95.00)	15 (29.41)	*χ*^2^ = 49.20	<0.05**
Ipsilateral tinnitus frequency (Hz)	4356.03 ± 2772.49	5886.55 ± 2760.17	*t* = 2.90	0.004**
Ipsilateral relative tinnitus loudness (dB SL)	10.03 ± 8.26	9.14 ± 5.21	*t* = 0.67	0.505
Opposite tinnitus frequency (Hz)	4603.68 ± 2766.00	5363.18 ± 3068.67	*t* = 1.37	0.173
Opposite relative tinnitus loudness (dB SL)	7.75 ± 9.31	5.25 ± 4.83	*t* = 1.73	0.087
HAMA	10.93 ± 3.64	10.33 ± 3.91	*t* = 0.84	0.404
HAMD	6.15 ± 2.02	6.37 ± 2.43	*t* = 0.53	0.600
PSQI	13.45 ± 4.34	13.33 ± 3.75	*t* = 0.15	0.881
THI	33.73 ± 13.00	32.33 ± 13.14	*t* = 0.56	0.575

Group 1 = non-hearing-loss group (NHL, *n* = 60); Group 2 = hearing-loss group (HL, *n* = 51), stratified according to the WHO 1997 classification of hearing impairment. Continuous variables are presented as mean ± SD; categorical variables as n (%). *t*: independent-samples *t*-test statistic; *χ*^2^: Chi-square statistic. HAMA = Hamilton Anxiety Rating Scale; HAMD = Hamilton Depression Rating Scale; PSQI = Pittsburgh Sleep Quality Index; THI = Tinnitus Handicap Inventory. **p < 0.05.

### Effects of the mixed acoustic intervention on tinnitus characteristics and psychometric outcomes

Repeated-measures ANOVA results are summarized in Table 2, and the simple-effect decomposition of significant interactions is presented in Table 3. Significant main effects of time were observed for tinnitus frequency (*F* = 6.49, *p* < 0.05, partial *η*^2^ = 0.056), relative loudness (*F* = 5.09, *p* < 0.05, partial η^2^ = 0.045), and PSQI score (*F* = 24.41, *p* < 0.05, partial *η*^2^ = 0.183), indicating significant post-treatment improvement in both groups. No significant Group × Time interactions were observed for these variables, indicating that the magnitude of improvement was similar between the HL and NHL groups.

**Table 2:** Repeated-measures ANOVA results before and after intervention for the two groups.

Variable	Effect	*F*	*p*	Partial *η*^2^
Ipsilateral tinnitus frequency	Group	6.41	0.013**	0.056
	Time	6.49	0.012**	0.056
	Group × Time	1.21	0.274	0.011
Ipsilateral relative loudness	Group	0.59	0.446	0.005
	Time	5.09	0.026**	0.045
	Group × Time	0.00	0.995	0.000
Opposite tinnitus frequency	Group	0.96	0.331	0.009
	Time	3.38	0.069	0.030
	Group × Time	1.02	0.314	0.009
Opposite relative loudness	Group	3.11	0.080	0.028
	Time	1.23	0.270	0.011
	Group × Time	0.21	0.646	0.002
HAMA	Group	0.15	0.698	0.001
	Time	14.26	< 0.001**	0.116
	Group × Time	4.13	0.044**	0.037
HAMD	Group	1.88	0.173	0.017
	Time	0.86	0.356	0.008
	Group × Time	1.09	0.300	0.010
PSQI	Group	1.24	0.268	0.011
	Time	24.41	< 0.001**	0.183
	Group × Time	3.14	0.079	0.028
THI	Group	0.88	0.351	0.008
	Time	22.89	< 0.001**	0.174
	Group × Time	5.96	0.016**	0.052

Main effect of Group: Group 1 (NHL) vs. Group 2 (HL). Main effect of Time: pre-vs. post-intervention. Interaction effect: Time × Group. *F*: F-statistic. **p < 0.05. Partial *η*^2^ = effect size (small ≈ 0.01, medium ≈ 0.06, large ≈ 0.14). HAMA = Hamilton Anxiety Rating Scale; HAMD = Hamilton Depression Rating Scale; PSQI = Pittsburgh Sleep Quality Index; THI = Tinnitus Handicap Inventory.

**Table 3:** Simple-effect analysis for the significant Group × Time interactions identified in Table 2 (HAMA and THI).

Variable	Stratum	Comparison	*t*	*p*
HAMA	Group 1 (NHL)	Pre vs. post	4.29	<0.001**
HAMA	Group 2 (HL)	Pre vs. post	1.19	0.238
HAMA	Pre-intervention	Group 1 vs. Group 2	0.74	0.461
HAMA	Post-intervention	Group 1 vs. Group 2	−1.40	0.163
THI	Group 1 (NHL)	Pre vs. post	5.33	<0.001**
THI	Group 2 (HL)	Pre vs. post	1.59	0.114
THI	Pre-intervention	Group 1 vs. Group 2	0.54	0.592
THI	Post-intervention	Group 1 vs. Group 2	−2.11	0.036**

Group 1 = NHL; Group 2 = HL. *t*: *t*-statistic. **p < 0.05. HAMA = Hamilton Anxiety Rating Scale; THI = Tinnitus Handicap Inventory.

**Figure 1:**
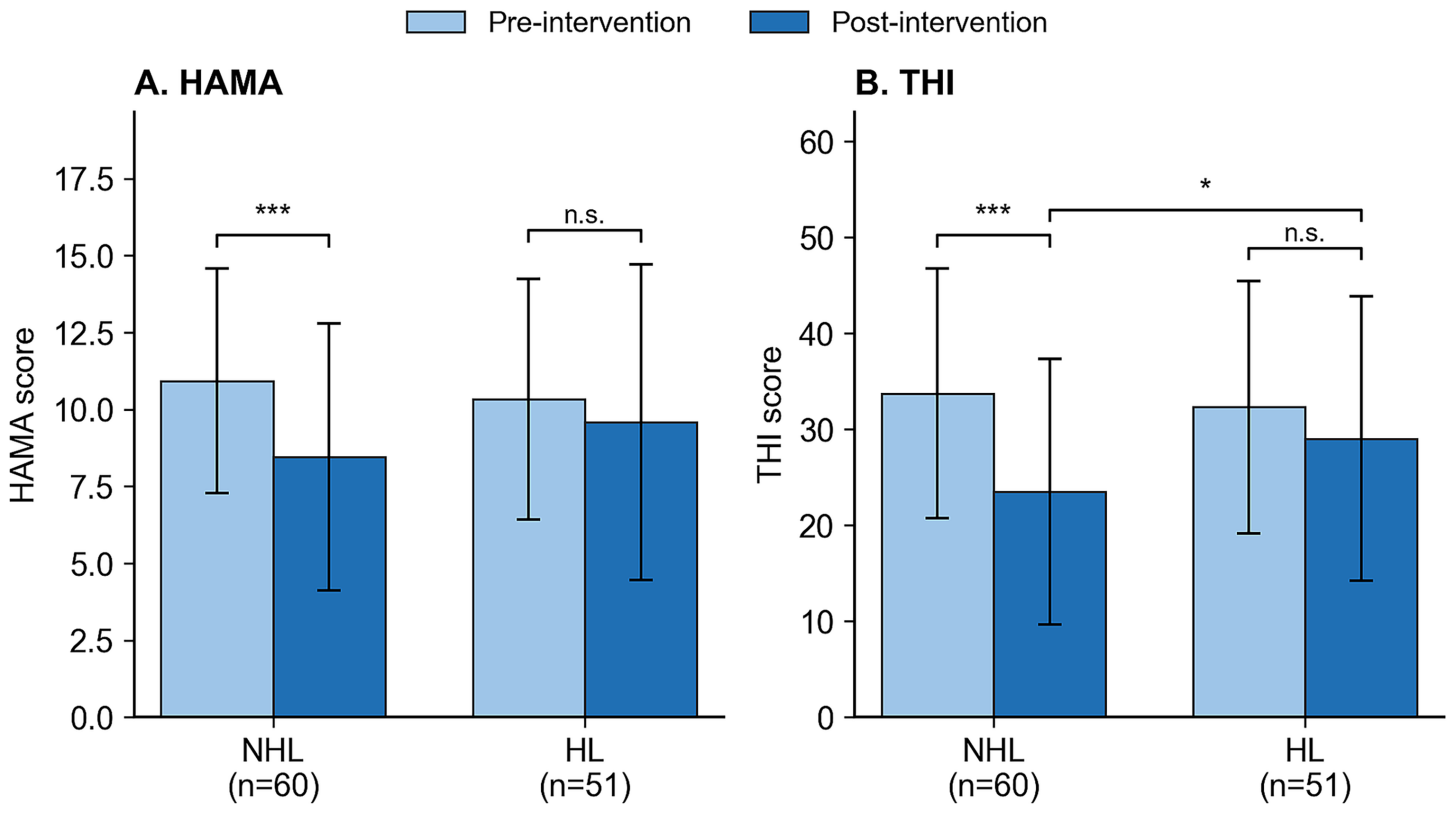
Pre- and post-intervention HAMA **(A)** and THI **(B)** scores in the non-hearing-loss (NHL, *n* = 60) and hearing-loss (HL, *n* = 51) groups after 3 months of personalized mixed acoustic therapy. Bars represent mean ± SD. Within-group pre-to-post comparisons (simple-effect analyses derived from the significant Group × Time interactions reported in Tables 2, 3): the NHL group showed significant reductions in both HAMA (*t* = 4.29, *p* < 0.001) and THI (*t* = 5.33, *p* < 0.001), whereas the HL group showed no significant change in either measure. After intervention, the NHL group had significantly lower THI scores than the HL group (*t* = −2.11, *p* = 0.036). HAMA = Hamilton Anxiety Rating Scale; THI = Tinnitus Handicap Inventory. ***p < 0.001; *p < 0.05; n.s. = not significant.

In contrast, significant Group × Time interactions were observed for HAMA (*F* = 14.26, *p* < 0.001, partial *η*^2^ = 0.116) and THI (*F* = 22.89, *p* < 0.001, partial *η*^2^ = 0.174). Simple-effect analyses (Table 3; Figure 1) showed that the NHL group had significantly greater reductions in both HAMA (*t* = 4.29, *p* < 0.001) and THI (*t* = 5.33, *p* < 0.001) than the HL group. Within the HL group, pre-to-post changes did not reach statistical significance for either HAMA (*t* = 1.19, *p* = 0.238) or THI (*t* = 1.59, *p* = 0.114), whereas within the NHL group both reductions were highly significant. After intervention, the NHL group also had significantly lower THI scores than the HL group (*t* = −2.11, *p* = 0.036).

### Predictors of favorable response to acoustic intervention

Binary logistic regression was used to identify baseline predictors of a favorable outcome (Table 4). NHL status was a significant predictor of favorable outcome (*β* = −1.97, *p* < 0.001; OR = 0.14, 95% CI 0.05–0.37, with HL as the reference category, indicating that NHL patients had higher odds of a favorable outcome than HL patients). Female sex (*β* = −1.46, *p* = 0.004; OR = 0.23, 95% CI 0.09–0.62), lower baseline PSQI score (*β* = 0.42, *p* < 0.001; OR = 1.53, 95% CI 1.21–1.93), and lower baseline HAMA score (*β* = −0.28, *p* = 0.016; OR = 0.76, 95% CI 0.60–0.95) were also independent predictors of a favorable outcome.

**Table 4:** Binary logistic regression for baseline predictors of a favorable response (THI reduction ≥7 points).

Variable	*β*	Wald	*p*	OR	95% CI
Hearing loss status (NHL vs. HL)	−1.97	15.47	<0.001**	0.14	0.05–0.37
Sex (female vs. male)	−1.46	8.42	0.004**	0.23	0.09–0.62
Baseline PSQI	0.42	12.78	<0.001**	1.53	1.21–1.93
Baseline HAMA	−0.28	5.82	0.016**	0.76	0.60–0.95

Reference category for “Hearing loss status”: Group 2 (HL). The negative β for “Hearing loss status” indicates that Group 1 (NHL) is associated with higher odds of a favorable outcome. OR = odds ratio; CI = confidence interval. **p < 0.05. *β* = regression coefficient; Wald = Wald *χ*^2^ statistic; PSQI = Pittsburgh Sleep Quality Index; HAMA = Hamilton Anxiety Rating Scale.

## Discussion

This study evaluated a personalized mixed acoustic therapy (narrow-band noise combined with music) in chronic tinnitus patients with and without hearing loss. Three main findings emerged. First, NHL patients were significantly younger and had higher prevalences of prior psychiatric disorders and insomnia than HL patients at baseline. Second, although both groups showed comparable improvements in tinnitus frequency, loudness, and sleep quality, the NHL group showed significantly greater reductions in anxiety (HAMA) and tinnitus-related disability (THI). Third, NHL status, female sex, and lower baseline anxiety and sleep disturbance were independent predictors of a favorable response.

The baseline differences we observed—younger age and higher rates of psychiatric and insomnia history in the NHL group—are consistent with growing evidence supporting phenotypic stratification in chronic tinnitus. This view distinguishes a “sensory/cochlear” subtype, driven by peripheral auditory pathology, from a “distress-associated” subtype, in which psychological factors predominate (22). Our data fit this framework: the HL group is likely to represent the sensory-driven subtype, whereas the NHL group displays features of the distress-predominant phenotype. This distinction is mechanistically important for interpreting the differential treatment response described below, in which the same intervention produced markedly different psychological benefits in the two phenotypes.

The pathophysiology of hearing-loss-associated tinnitus is well explained by the “central gain” hypothesis. Following cochlear injury, reduced auditory input triggers compensatory upregulation of central gain, leading to increased spontaneous neural firing. Elevated firing rates and aberrant neural synchrony in the auditory cortex and dorsal cochlear nucleus underlie tinnitus (23). Chronic tinnitus following noise-induced hearing loss is associated with increased gamma-band oscillations in the auditory cortex, a biomarker of pathological neural synchrony (24).

In contrast, recent work on tinnitus without hearing loss emphasizes the role of central sensitization and functional changes in non-auditory networks. Resting-state functional magnetic resonance imaging (fMRI) has shown increased functional connectivity between the default mode network (DMN) and the salience network in patients without hearing loss, and this connectivity correlates with emotional distress scores (25). These findings suggest that in NHL patients, tinnitus may be sustained by a brain state characterized by excessive self-referential processing and heightened attention to the phantom sound. Accumulating evidence also indicates that the limbic system—particularly the amygdala and anterior cingulate cortex (ACC)—acts as an “emotional amplifier” that intensifies tinnitus perception even in the absence of peripheral damage (26). The higher prevalence of psychiatric comorbidity in our NHL cohort therefore likely reflects intrinsic dysregulation of limbic and prefrontal circuits that is part of the tinnitus pathophysiology itself, rather than a secondary consequence.

The comparable reductions in tinnitus frequency and loudness in both groups indicate that the intervention modulates primary auditory processing largely independently of hearing status. Personalized acoustic stimulation may reduce tinnitus perception through several mechanisms, including lateral inhibition—in which spectral energy adjacent to the tinnitus frequency suppresses hyperactive neural ensembles—and stochastic resonance, which reduces the signal-to-noise contrast between tinnitus-related and background neural activity (27). Electrophysiological studies have shown that sound-based interventions can reduce abnormal neural synchrony along the auditory pathway, an effect that appears to be largely independent of peripheral hearing status (28).

However, the divergent psychological benefits suggest a more complex top-down mechanism, mediated primarily by the musical component of the intervention. Music is a potent modulator of emotion and arousal. Neuroimaging consistently shows that pleasant music activates mesolimbic reward circuitry—including the nucleus accumbens (NAc)—triggering dopamine release and inducing positive affect (29). An fMRI study in tinnitus patients reported that music listening reduced amygdala reactivity and strengthened connectivity between the auditory cortex and the ventromedial prefrontal cortex (vmPFC), a key region for emotional regulation (30). Music also reduces sympathetic arousal and increases parasympathetic activity, thereby alleviating anxiety (31).

In NHL patients, an intact peripheral auditory system enables faithful transmission of musical features (melody, harmony, rhythm), allowing full engagement of these emotional regulation networks. The positive affect induced by music may facilitate cognitive reappraisal of tinnitus, helping patients reinterpret the phantom sound as less threatening—a process central to cognitive-behavioral models of tinnitus distress (32). NHL patients can therefore effectively integrate this pleasurable auditory signal, leading to greater psychological benefit.

In contrast, HL patients receive degraded acoustic input. Sensorineural hearing loss impairs the processing of temporal fine structure and frequency resolution, distorting the perception of musical consonance and valence (33). The reward potential of music is therefore reduced, and neural responses in the NAc and vmPFC may be suboptimal. Hearing loss may also reinforce maladaptive connections between the auditory pathway and the limbic system (e.g., between the dorsal cochlear nucleus and the amygdala), establishing a persistent negative emotional association with auditory stimuli (34). This “limbic lock-in” may prevent positive emotional signals from degraded music from overriding established negative tinnitus schemas, limiting psychological improvement.

Our logistic regression model identified NHL status, female sex, and lower baseline anxiety and sleep disturbance as independent predictors of a favorable outcome, providing a basis for clinical decision-making. The association between NHL status and better psychological outcomes is consistent with the mechanisms outlined above: patients with preserved hearing can more fully benefit from the emotional modulation provided by the musical component. The predictive value of female sex is consistent with existing literature on sex differences in tinnitus treatment response. Partyka et al. reported that acoustic stimulation produced gender-differentiated effects in tinnitus patients, with women showing distinct response patterns in affective processing—possibly reflecting sex-related differences in interhemispheric connectivity and in hormonal modulation of mood-related neurotransmitter systems (5). Lower baseline anxiety and sleep disturbance may indicate less entrenched limbic–tinnitus coupling, allowing the therapy to engage adaptive emotional processes more effectively (35).

These findings have several clinical implications.

### Pre-treatment stratification

Baseline assessment should include not only audiometry but also validated measures of anxiety, depression, sleep quality, and tinnitus-related disability. This allows clinicians to identify likely responders—for example, younger NHL women with moderate baseline anxiety—who may derive the greatest benefit from this combined therapy.

### Realistic expectation setting

For patients with substantial hearing loss or severe baseline anxiety or insomnia, clinicians should clearly discuss the expected pattern of response: a potential reduction in tinnitus loudness, but limited improvement in overall emotional distress. A multimodal strategy is appropriate in these cases. For patients with severe anxiety or insomnia, integrating Cognitive Behavioral Therapy for Insomnia (CBT-I) or CBT for Tinnitus with pharmacological management (e.g., selective serotonin reuptake inhibitors, sedative-hypnotics) may be necessary (36, 37). For patients with hearing loss, concurrent auditory rehabilitation with optimized hearing aids or cochlear implants may restore acoustic fidelity and thereby enable the emotional benefits of the therapy (38).

### Protocol optimization

For the identified optimal subgroup—NHL women with significant psychological burden—this mixed acoustic therapy may serve as a first-line intervention, targeting both auditory and emotional dimensions of tinnitus in a single non-pharmacological treatment.

A key methodological feature of this study is the systematic use of bilateral subjective tinnitus acoustic testing. The protocol measures perceived tinnitus frequency and loudness in each ear separately, regardless of how the patient subjectively localizes the tinnitus (unilateral, bilateral, or central). This addresses a common clinical challenge: the inclusion and systematic evaluation of patients who cannot reliably distinguish between ear-localized tinnitus and centrally perceived “head noise.” By capturing individualized acoustic profiles in this way, the method broadens cohort inclusivity and provides objective baselines for tailoring the narrow-band noise component and for tracking treatment-induced changes in the auditory domain.

## Limitations and future directions

Several limitations should be acknowledged. *First*, this is a non-randomized observational cohort study without a control or sham acoustic condition. Chronic tinnitus is known to undergo spontaneous fluctuation, demonstrate substantial placebo responsiveness, and exhibit regression to the mean in repeated assessments. These confounding phenomena may have contributed to the observed pre-to-post improvements, particularly the equivalent gains seen in both groups for psychoacoustic parameters and sleep quality. The magnitude of the treatment-specific effect therefore cannot be unambiguously isolated, and future randomized controlled trials incorporating sham acoustic arms or active comparators (e.g., notched music alone) are required to establish causal efficacy. *Second*, our exclusion of patients with hypertension, diabetes mellitus, and coronary artery disease—conditions highly prevalent in the 40–60-year age range—may have introduced a selection bias that limits generalizability to real-world tinnitus clinic populations, where such comorbidities are the norm rather than the exception. *Third*, the 3-month follow-up is comparatively brief; given that tinnitus is a chronic condition characterized by frequent fluctuation, longer-term follow-up (12–24 months) is required to assess durability of the observed effects. *Fourth*, our mechanistic explanations remain speculative in the absence of direct neuroimaging (fMRI, EEG) or electrophysiological data (MEG) from this cohort to validate hypothesized differences in DMN and limbic network activity between groups. *Fifth*, the restricted age range (40–60 years) limits the applicability of these findings to younger or older demographics.

Future studies should address these gaps. Long-term follow-up (12–24 months) is needed to assess relapse rates and durability of treatment effects, and broader age criteria will improve external validity. Combining multimodal neuroimaging with longitudinal designs—for example, resting-state fMRI to track changes in DMN and salience-network connectivity, together with EEG to quantify auditory cortical oscillations (e.g., gamma-band power)—could link physiological changes to clinical outcomes (39). Combination strategies also warrant investigation: in HL patients, evaluating acoustic therapy together with advanced hearing aids (with wide dynamic range compression and noise management) or cochlear implants could test whether restoring acoustic fidelity enhances emotional regulation. Bimodal stimulation paradigms (e.g., combined auditory and somatosensory input) also warrant evaluation in this subgroup (40).

## Conclusion

In summary, personalized mixed acoustic therapy combining narrow-band noise and music reduced core psychoacoustic features of chronic tinnitus and improved sleep quality in patients both with and without hearing loss. Its effect on psychological burden—specifically anxiety and tinnitus-related disability—was significantly greater in patients without hearing loss. The independent predictors of a favorable outcome—NHL status, female sex, and lower baseline anxiety and sleep disturbance—illustrate the heterogeneity of chronic tinnitus and argue against a uniform treatment approach. These findings support a stratified, precision-medicine framework in which treatment decisions are guided by individual audiological and psychometric characteristics, and indicate that combining neuroimaging-informed mechanistic studies with multimodal interventions is a promising direction for translating this understanding into more effective personalized care.

## Data Availability

The original contributions presented in the study are included in the article/supplementary material, further inquiries can be directed to the corresponding author.
